# High Dimensional Mass Cytometry Analysis Reveals Characteristics of the Immunosuppressive Microenvironment in Diffuse Astrocytomas

**DOI:** 10.3389/fonc.2020.00078

**Published:** 2020-02-04

**Authors:** Weilun Fu, Wenjing Wang, Hao Li, Yuming Jiao, Jiancong Weng, Ran Huo, Zihan Yan, Jie Wang, Hongyuan Xu, Shuo Wang, Jiangfei Wang, Dexi Chen, Yong Cao, Jizong Zhao

**Affiliations:** ^1^Department of Neurosurgery, Beijing Tiantan Hospital, Capital Medical University, Beijing, China; ^2^China National Clinical Research Center for Neurological Diseases, Beijing, China; ^3^Institute of Hepatology, Capital Medical University Affiliated Beijing You'an Hospital, Beijing, China

**Keywords:** diffuse astrocytoma, oligodendroglioma, CyTOF, immune profiling, microenvironment

## Abstract

The tumor immune microenvironment (TIME) plays a pivotal role in tumor development, progression, and prognosis. However, the characteristics of the TIME in diffuse astrocytoma (DA) are still unclear. Leveraging mass cytometry with a panel of 33 markers, we analyzed the infiltrating immune cells from 10 DA and 4 oligodendroglioma (OG) tissues and provided a single cell-resolution landscape of the intricate immune microenvironment. Our study profiled the composition of the TIME in DA and confirmed the presence of immune cells, such as glioma-associated microglia/macrophages (GAMs), CD8+ T cells, CD4+ T cells, regulatory T cells (Tregs), and natural killer cells. Increased percentages of PD-1+ CD8+ T cells, TIM-3+ CD4+ T cell subpopulations, Tregs and pro-tumor phenotype GAMs substantially contribute to the local immunosuppressive microenvironment in DA. DAs and OGs share similar compositions in terms of immune cells, while GAMs in DA exhibit more inhibitory characteristics than those in OG.

## Introduction

Diffuse astrocytomas (DAs) account for 10% of all adult primary brain tumors (1). They are diffusely infiltrating World Health Organization (WHO) grade II brain neoplasms, and DA patients have a median survival in the range of 5–7 years (2). Even with a combination of available therapeutic modalities, including surgery, radiotherapy, and chemotherapy, the invasive growth and resistance to therapy exhibited by these tumors result in their recurrence, malignant transformation, and almost invariable progression to high-grade glioma in most patients (3). These challenges underscore the need for novel strategies to improve the outcomes of patients with low-grade glioma (LGG) (4).

Immunotherapy is an emerging breakthrough approach that promises the possibility of highly specific and less toxic treatment compared to conventional chemotherapy (5); this approach aims to induce an adaptive immune response that specifically targets and kills tumor cells without affecting normal cells. Thanks to advances in the fields of neuro- and cancer-immunology, a wide range of immunotherapies for WHO grade IV glioblastoma are now undergoing development, including antibodies, adoptive cell transfers, vaccines, virally-based treatments and immune checkpoint blockade (6–9). However, the efficacy of immunotherapy for the treatment of DAs is still controversial.

The infiltration of diverse immune cell populations has been reported in various cancer types, and the cooperation between tumor cells and tumor-infiltrating immune cells drives tumor development (10). Glioma cells secrete numerous cytokines, chemokines, and growth factors that promote the infiltration of a range of immune cells, such as resident microglia, peripheral macrophages, CD4+ T cells, CD8+ T cells, and regulatory T (Treg) cells, into the tumor (11–13), and these non-neoplastic cells play crucial roles in cancer growth, metastasis, and response to treatment. Therefore, sound knowledge of the immune microenvironment of DA will aid the design of effective therapeutic strategies and provide a foundation for the success of immunotherapy (14). In previous studies, histopathological analysis, immunohistochemistry, and flow cytometry were utilized to reveal the immunological features of the glioma immune microenvironment (15, 16). To the best of our knowledge, immune changes in the microenvironment of DA have rarely been reported, and a comprehensive understanding of the phenotypic characterization of immune cells in the DA tumor microenvironment at the protein level is highly needed.

To this end, we utilized mass cytometry (CyTOF) to examine the TIME of DAs and paired peripheral blood mononuclear cells (PBMCs). We also collected specimens of oligodendroglioma (OG) to compare the TIME in DAs and OGs. CyTOF enables the simultaneous measurement of more than 30 parameters per single cell using metal isotope-conjugated antibodies with minimal overlap, which maximizes the information obtained from each individual sample (17). By addressing the cellular and molecular complexity of the immunosuppressive microenvironment, our data provide a detailed dissection of the DA immune cell types and reveal immunosuppressive changes in glioma-associated microglia/macrophages (GAMs) and T cell exhaustion in DA lesions. Our data show that immunosuppressive programs are present in early stages in LGG and likely compromise antitumor immunity. Our study suggests that neoadjuvant immunotherapy strategies targeting innate immune cells in DA lesions have the potential to reactivate the TIME and transform the tumor response to affect checkpoint blockade.

## Materials and Methods

### Human Specimens

Blood and LGG tissues were obtained from patients with WHO grade II DA and OG undergoing craniotomy surgery at Beijing Tiantan Hospital (Beijing, China) from June 2018 to April 2019. All patients were diagnosed with WHO grade II diffuse DA or OG, which was confirmed by histopathology. None of the patients used glucocorticoids before sampling.

### Ethics Approval and Consent to Participate

This study was approved by the Institutional Review Board and Ethics Committee of Beijing Tiantan Hospital, Capital Medical University. Written informed consent was obtained from each patient.

### Glioma Tissue Single Cell Dissociation

DA or OG tissues were washed with ice-cold Dulbecco's phosphate-buffered saline (DPBS, without Mg^2+^ and Ca^2+^, catalog no. D8537, Sigma-Aldrich) immediately after surgery. Briefly, the DA or OG tissues were dissociated using type IV collagenase (catalog no. 17104019, GIBCO) for 10 min at 37°C. Then, the samples were washed with Dulbecco's modified Eagle medium (DMEM, catalog no. D5796, Sigma-Aldrich) and centrifuged at 300 g for 4 min at 18°C with minimal braking. The samples were then filtered through a 40 mm cell strainer with DPBS and washed with red blood cell (RBC) lysis buffer (catalog no. 555899, BD Biosciences). The dissociated cell suspension was then washed twice with DPBS. The cell pellet was resuspended in staining buffer (DPBS containing 5% fetal bovine serum, FBS; catalog no. 0500, ScienCell).

### Blood Sample Single Cell Dissociation

Fresh blood samples were collected into ethylenediaminetetraacetic acid (EDTA) anticoagulation tubes and then centrifuged at 800 g for 5 min with minimal braking to remove the plasma. Then, the samples were transferred into SepMate PBMC isolation tubes containing Ficoll (catalog no. 86450, STEMCELL Technologies) and centrifuged at 1,200 g for 10 min with minimal braking. The cells were washed with RBC lysis buffer. Then, the cells were washed twice with DPBS and resuspended in staining buffer.

### Mass Cytometry

A panel of 33 antibodies designed to distinguish a broad range of immunocytes was used. Antibodies were either purchased in a pre-conjugated form from Fluidigm or purchased in a purified form from Biolegend and conjugated in-house using the Maxpar® X8 Multimetal Labeling Kit (catalog no. 201300, Fluidigm) according to the manufacturer's recommendations. The antibodies and reporter isotopes are listed in Table S1. Briefly, the cell samples were rewarmed rapidly. Cells from glioma tissue were stained with anti-CD45 antibody conjugated with 156Gd, while cells from PBMCs were first stained with anti-CD45 antibody conjugated with 89Y. Then, glioma and PBMC cells were mixed together and stained with cell surface antibodies for 30 min at room temperature. Subsequently, the samples were permeabilized overnight at 4°C and stained with intracellular antibodies for 30 min at room temperature. The antibody-labeled samples were washed and incubated in 0.125 nM intercalator-Ir (catalog no. 201192B, Fluidigm) diluted in phosphate-buffered saline (PBS, catalog no. 806544, Sigma-Aldrich) containing 2% formaldehyde and stored at 4°C until CyTOF examination. Before acquisition, the samples were washed with deionized water and then resuspended at a concentration of 1 ×10^6^ cells/mL in deionized water containing a 1:20 dilution of EQ Four Element Beads (catalog no. 201078, Fluidigm). The samples were then examined by mass cytometry (Fluidigm).

### CyTOF Data Analysis

Data were obtained as.fcs files. The addition of EQ Four Element Beads allowed us to use a MATLAB-based normalization technique utilizing bead intensities as previously described (18). The CyTOF data were analyzed with Cytobank (www.cytobank.org). The cell types were identified based on the following parameters: T cells, CD45+ CD3+; natural killer (NK) cells, CD45+ CD3-CD16+ CD56+ (10, 19); B cells, CD45+ CD19+; monocytes, CD45+ CD14+ CD16+ (20); macrophages or microglial cells, CD45+ CD11b+ CD3-CD19-CD66b- (15); Tregs, CD45+ CD4+ CD25+ CD127- (21), and granulocytes, CD45+ CD66b+. Monocytes and macrophages constitute mononuclear phagocytes (22). Manual gating was applied to indicate the cell types as previously reported (23). ViSNE (24) algorithms were used on the indicated gated cells. The viSNE analysis of T cells or GAMs was performed for patients with samples with more than 500 cell counts for both PBMCs and tumor lesions. Then, the automatic cluster gate functionality was used for the hierarchical cluster analysis. Heatmaps were generated by R software (version 3.4.0).

### Heatmap Data Normalization

For **Figures 3D**, **4C**, the log10-scaled values were used.

For **Figures 3E,F**, we calculated the ratio of the value of each T cell cytokine or marker to that of the paired PBMC T cells in each patient and then calculated the log10-scaled ratio to obtain the normalized values.

### Immunohistochemistry and Immunofluorescence

DA samples were fixed overnight at 4°C in 4% formalin and embedded in paraffin blocks to obtain paraffin sections. Immunohistochemical staining was performed as previously reported (25). For immunofluorescence, 3 μm paraffin sections were washed twice in PBS (catalog no. 806544, Sigma-Aldrich) for 15 min, permeabilized in 0.2–0.5% Triton X-100 (catalog no. T8200-100, Solarbio) and blocked in 5% normal donkey serum (catalog no. 017-000-001, Jackson Lab) for 1 h and stained with primary antibody overnight. The primary antibodies were detected using fluorescent-conjugated secondary antibodies (catalog no. PV-6000, ZSGB-BIO). Sections were mounted with fluorescence mounting medium (catalog no. S3023, Dako). As previously reported (26), the Opal 4-Color Manual IHC Kit (catalog no. NEL810001KT, Perkin Elmer) was used for the analysis of the formalin-fixed paraffin-embedded DA sections according to the manufacturer's protocol. Fluorescent images were acquired with a Zeiss LSM880 NLO microscope. The primary antibodies were anti-CD45 (catalog no. AB40763, Abcam), anti-CD11b (catalog no. 21851-1-AP, Proteintech), anti-TNFα (catalog no. 60291-1-Ig, Proteintech), and anti-IDO (catalog no. 86630S, CST).

### Statistics

For the CyTOF experiments, 10 DA samples and paired PBMCs and 4 OG samples were analyzed. The Wilcoxon matched-pair signed rank test and Mann–Whitney test were used accordingly to analyze the statistical significance. The statistical analysis was performed using GraphPad Prism (version 7.00). *P* < 0.05 were considered statistically significant.

### Data Availability

The raw CyTOF data used and analyzed in the current study are available from the corresponding author upon reasonable request.

## Results

### Single-Cell Profiling of the Diffuse Astrocytoma Immune Microenvironment

We obtained 10 WHO grade II DAs and paired peripheral blood samples as well as 4 OG tumor tissues. The baseline characteristics of all patients are summarized in Table 1.

**Table 1:** Basic characteristics of all patients.

**No**.	**Histopathology**	**Age**	**Gender**	***IDH1***	***IDH2***	***1p19q***	***TERT* promoter**
0759	DA	39	Male	Mut	Wt	Noncodel	Wt
0884	DA	41	Male	Mut	Wt	Noncodel	Wt
1827	DA	52	Female	Mut	Wt	Noncodel	C228T
8974	DA	67	Female	Mut	Wt	Noncodel	C250T
9144	DA	38	Male	Mut	Wt	Noncodel	C250T
9852	DA	38	Female	Wt	Wt	Noncodel	Wt
1837	DA	36	Male	Mut	Wt	Noncodel	Wt
5189	DA	34	Male	Mut	Wt	Noncodel	Wt
5749	DA	52	Female	Mut	Wt	Noncodel	Wt
7684	DA	28	Male	Mut	Wt	Noncodel	Wt
9203	OG	36	Male	Mut	Wt	Codel	Wt
7541	OG	49	Male	Wt	Wt	Codel	C250T
2948	OG	30	Male	Mut	Wt	Codel	C228T
5749	OG	52	Female	Mut	Wt	Codel	Wt

*DA, diffuse astrocytoma; OG, oligodendroglioma; IDH, isocitrate dehydrogenase; TERT, telomerase reverse transcriptase; Wt, wild type; Mut, mutation, Codel, codeletion*.

**Figure 1:**
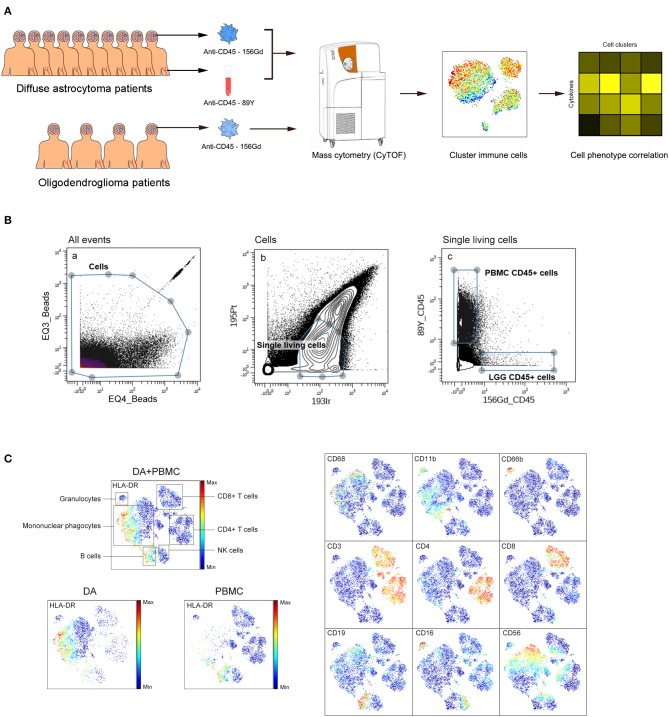
Analysis of the immunosuppressive microenvironment of DA using mass cytometry. **(A)** Schematic defining the immune composition of DAs and OGs. Glioma tissues and paired PBMC samples were collected from patients. Samples were processed and stained with antibodies conjugated to metal isotopes. CyTOF single cell data were used to identify the immune features of patients. **(B)** All ungated events were sequentially gated in Cytobank to identify CD45+ cells. (a) EQ3 beads and EQ4 beads were used to identify cells. (b) Single living cells were identified by gating the cells negative for 195Pt and positive for 193Ir. (c) CD45+ cells from LGG PBMCs were obtained from living single cells. **(C)** ViSNE plots of complete immune systems according to the relative expression of CyTOF markers in all samples. The cell populations are also indicated (left). Five hundred immune cells per sample were included in the viSNE analysis.

We simultaneously mapped the immune compartments of DA, OG lesions, and PBMCs (Figure 1A). The initial gating strategies used for CD45+ cells are provided in Figure 1B, and the gating strategies used for the indicated immune cells are summarized in Table S2. The ViSNE map of CD45 + cells collected from all DA samples showed differential abundances of infiltrating immune cell populations in the DA immune microenvironment compared to those in peripheral blood (Figure 1C).

### Mononuclear Phagocytes and T Cells Dominate the Diffuse Astrocytoma Immune Microenvironment

**Figure 2:**
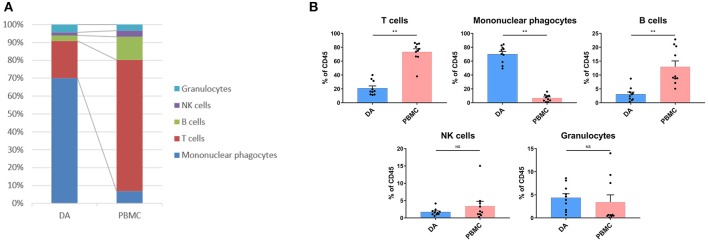
Immunosuppressive changes in the DA microenvironment. **(A)** Composition of the CD45+ compartment showing the average frequencies of the major immune lineages in each tissue. **(B)** Bar plots showing the frequencies for each DA patient and paired PBMC sample (by Wilcoxon matched-pair signed rank test). Bar plots show the mean with the SEM (NS, no significance; **p < 0.01).

We analyzed the distributions of the different immune cell lineages that accumulated in DAs and paired PBMCs in patients. The most abundant immune cells in the DA immune microenvironment were mononuclear phagocytes (70.02%) and T lymphocytes (20.86%). Compared with that in PBMCs, the proportion of mononuclear phagocytes was significantly increased in DAs (*p* < 0.01), while the proportions of T cells and B cells were significantly decreased (*p* < 0.01), and the proportions of NK cells and granulocytes were similar (Figures 2A,B).

### T Cells Are Exhausted, and Tregs Are Increased in the Diffuse Astrocytoma Immune Microenvironment

**Figure 3:**
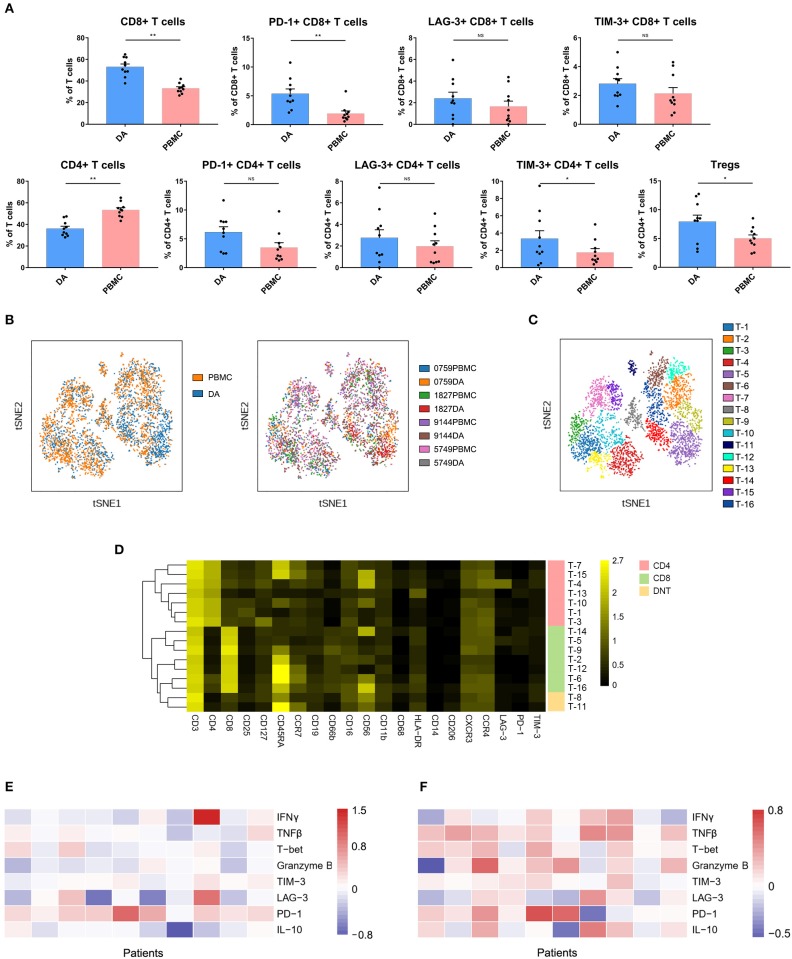
Exhausted T cell compartment in DA lesions. **(A)** Bar plots showing the frequencies of T cell subgroups in tumor sites and PBMCs from patients with DA (by Wilcoxon matched-pair signed rank test). Bar plots show the mean with the SEM (NS, no significance; *p < 0.05, **p < 0.01). **(B)** ViSNE map, colored by sample type (left) or sample source (right), displaying T cell subgroups in 4 patients. **(C)** ViSNE map, colored by clusters, displaying T cell subgroups in 4 patients. **(D)** Heatmap showing the normalized expression of the indicated markers for 16 T cell clusters identified in the 4 patients. **(E,F)** Heatmap showing relative marker expression levels in four DA patients. The relative marker expression levels were determined by the ratios of the indicated marker expression levels of T cells in tumor sites to those in PBMCs.

Compared with that in PBMCs, the percentage of CD4+ T cells (*p* < 0.01) was decreased, while that of CD8 + T cells (*p* < 0.01) was increased in DAs. Specifically, the Treg proportion in the DA lesions was significantly increased in all patients (*p* < 0.05) (Figure 3A). Programmed cell death protein 1 (PD-1)-, T cell immunoglobulin domain and mucin domain-3 (TIM-3)- or lymphocyte activation gene 3 (LAG-3)-positive T cells are recognized as exhausted subsets (27–29). Compared to those in PBMCs, the proportions of TIM-3+ CD4+ T cells (*p* < 0.05) and PD-1+ CD8+ T cells (*p* < 0.01) were remarkably higher in tumor sites (Figure 3A).

The dimensionality reduction tool viSNE (24) was employed to convert the high-dimensional CyTOF data from each sample into a two-dimensional map. Among the 10 DA patients, four patients had more than 500 T cells in both the tumor lesions and the PBMCs, and viSNE analysis was performed for these patients. In the viSNE map, T cells in tumor sites displayed similar distributions to those in PBMCs (Figure 3B). A hierarchical cluster analysis of the T cells using the automatic cluster gate functionality was performed to fully capture the heterogeneity of the T cell compartment. According to the surface markers, the T cells were subdivided into 16 subgroups (Figure 3C). The expression profiles of the T cell clusters were visualized in a heatmap (Figure 3D). This approach led to the identification of seven CD4+ phenotypes, seven CD8+ phenotypes and two CD4+/CD8+ double-negative phenotypes.

Although the CD8+ T cell proportion was elevated in tumor sites, their ability to secrete the antitumor cytokines interferon γ (IFNγ), tumor necrosis factor β (TNFβ), T-bet and granzyme B was reduced compared to that of the CD8+ T cells in the PBMCs, while PD-1 was more frequently expressed on CD8+ T cells in PBMCs (Figure 3E). Compared to those on CD4+ T cells in PBMCs, the expression levels of antitumor (TNFβ, T-bet, and granzyme B) and protumor (PD-1 and IL-10) markers on CD4+ T cells in tumor sites were commonly higher (Figure 3F).

### Glioma-Associated Microglia/Macrophages Were Clearly Distinguishable From Mononuclear Phagocytes in PBMCs

**Figure 4:**
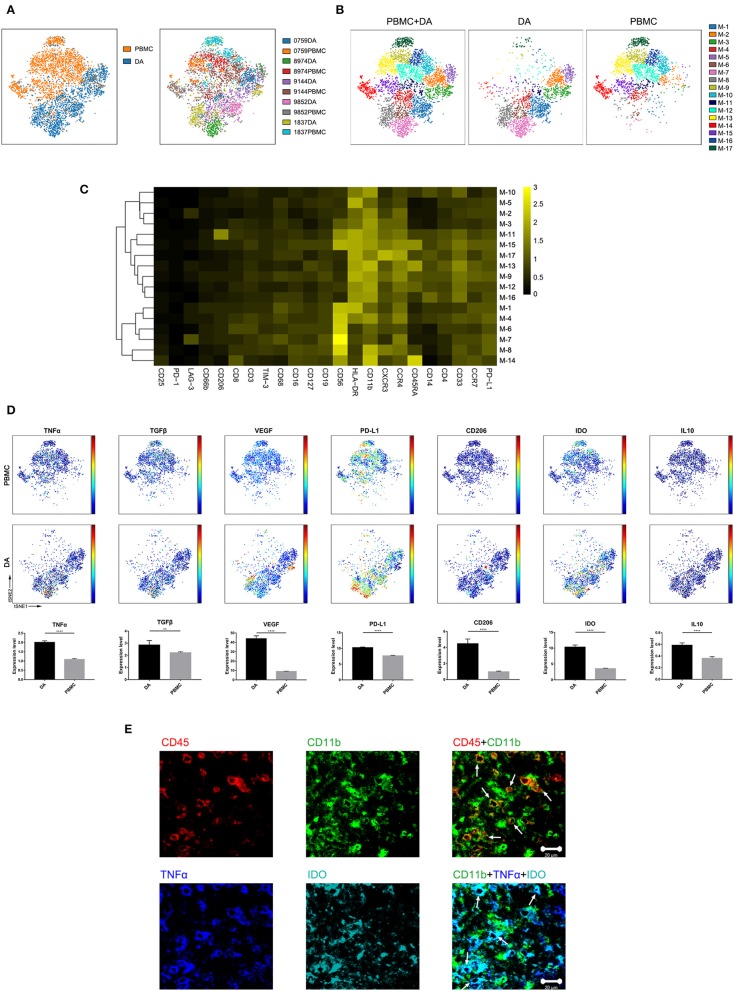
Characterization of GAM phenotypes in DA. **(A)** ViSNE map, colored by sample type (left) or sample source (right), displaying GAM subgroups in five patients. **(B)** ViSNE map, colored by clusters, displaying the GAM subgroup distribution in DA lesions and PBMCs. **(C)** Heatmap showing the normalized expression of the indicated markers for 17 GAM clusters identified in five patients. **(D)** Normalized expression of the indicated markers on the viSNE map. Bar plots show significant differences in the expression levels of the indicated markers between PBMCs and DA lesions (by the Mann–Whitney test). Bar plots show the mean with the SEM (**p < 0.01; ****p < 0.0001; NS, no significance). **(E)** Representative DA tissue stained for CD11b (green), CD45 (red), IDO (cyan), and TNFα (blue). Costaining of CD45 and CD11b (upper) indicated that most CD45+ immunocytes in DA were CD11b+ cells. Costaining of CD11b, IDO, and TNFα (lower) demonstrated that GAMs could coexpress TNFα and IDO (arrows).

Previous studies showed the extensive infiltration of gliomas with peripheral macrophages and resident microglia (30), which are collectively termed GAMs. In the current study, GAMs were the most enriched population in DA lesions. Five patients had more than 500 GAM cells or mononuclear phagocytes in both tumor sites and PBMCs, and viSNE analysis was performed on these cells. The ViSNE plot showed that GAMs were clearly distinguishable from mononuclear phagocytes in PBMCs (Figure 4A). According to the surface markers, GAMs or mononuclear phagocytes could be subdivided into 17 subgroups, with 6 subgroups mainly resident in DA lesions, 8 subgroups mainly resident in PBMCs, and 3 existing in both tumor sites and PBMCs (Figure 4B). The expression profiles of the GAM clusters were visualized in a heatmap (Figure 4C).

The viSNE map showed the elevated expression of both the anti-tumor marker tumor necrosis factor α (TNFα) and the pro-tumor markers transforming growth factor β (TGFβ), vascular endothelial growth factor (VEGF), programmed death-ligand 1 (PD-L1), CD206, indoleamine-pyrrole 2,3-dioxygenase (IDO), and IL10 in GAMs compared with those in mononuclear phagocytes in PBMCs (Figure 4D). A subgroup of GAMs represented in cluster M-8, which mainly existed in DA lesions, displayed high levels of VEGF and PD-L1 expression. GAMs may promote T cell apoptosis through expressing PD-L1 (31, 32). By secreting VEGF, GAMs might differentiate into a pro-angiogenic and immunosuppressive phenotype (26). Meanwhile, certain GAM subgroups (M-7) could coexpress antitumor (TNFα) and protumor (IDO and PD-L1) markers.

We revealed mononuclear macrophage infiltration in DA lesions using immunohistochemical and immunofluorescence costaining and verified the finding that antitumor (TNFα) and protumor (IDO) markers were coexpressed in certain GAM subgroups (Figure 4E).

### Natural Killer Cells Are Not Cytolytic in Diffuse Astrocytoma Lesions

**Figure 5:**
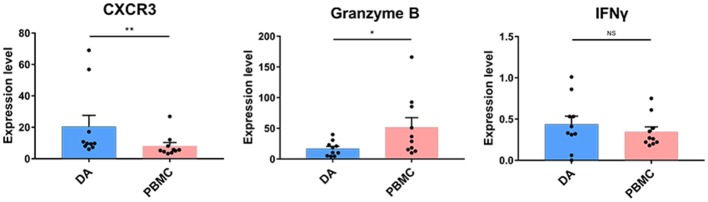
Cytolytic NK cells are dysregulated at the tumor site. Bar plots showing CXCR4, granzyme B, and IFNγ expression in NK cells from patients with DA and paired PBMCs (by the Wilcoxon matched-pair signed rank test). Bar plots show the mean with the SEM (*p < 0.05; **p < 0.01; NS, no significance).

NK cell proportions were not significantly increased at the tumor site compared with those in the peripheral blood of patients, although the NK cells that infiltrated into the tumor lesions expressed higher levels of CXCR3 (*p* < 0.01) (Figure 5), which is a molecule reported to be required for NK cell infiltration (33). Moreover, the NK cells that remained at the tumor site showed lower levels of cytolytic activity, as these cells expressed similar levels of IFNγ and lower levels of granzyme B compared to those in peripheral blood (Figure 5).

### The Tumor Immune Microenvironment of Diffuse Astrocytoma Exhibits More Inhibitory Characteristics Than That of Oligodendroglioma

**Figure 6:**
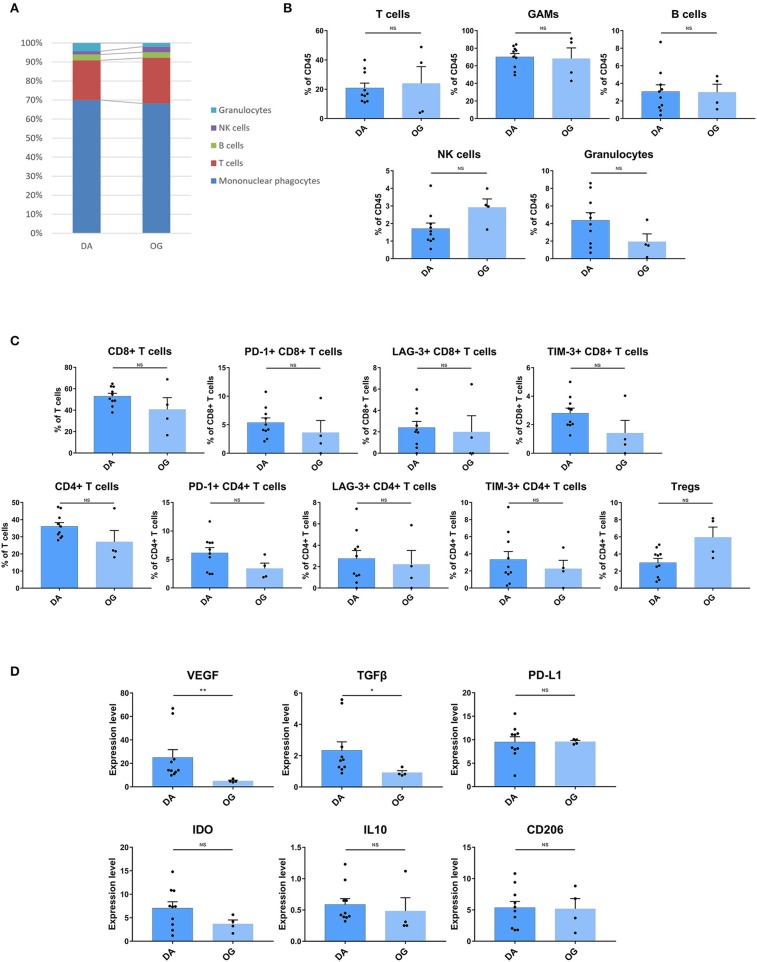
The TIME of DA shows more inhibitory characteristics than that of OG. **(A)** The frequencies of DA and OG immunocytes. Composition of the CD45+ compartment showing the average frequencies of the major immune lineages in each tissue. **(B)** Bar plots showing the frequencies for each DA patient and OG patient (by the Mann–Whitney test). Bar plots show the mean with the SEM (NS, no significance). **(C)** Bar plots showing the frequencies of T cell subgroups in DA and OG (by the Mann–Whitney test). Bar plots show the mean with the SEM (NS, no significance). **(D)** Bar plots of pro-tumor marker expression in GAMs in DA and OG (by the Mann–Whitney test). Bar plots show the mean with the SEM (*p < 0.05; **p < 0.01; NS, no significance).

The composition of immune cell subsets was similar in the DAs and OGs (Figures 6A,B). The proportions of the T cell subpopulations in DAs and OGs were also similar, and T cells in DAs and OGs demonstrated comparable exhaustion trends (Figure 6C). The pro-tumor markers TGFβ and VEGF were more strongly expressed by GAMs in DAs than in OGs, while IL10, PD-L1, CD206, and IDO were similarly expressed by GAMs in DA and OGs (Figure 6D).

## Discussion

The TIME in DAs plays essential roles in tumor development, progression, and prognosis. Comprehensive profiling of the intricate milieu and its interactions remains lacking, and single-cell technologies such as CyTOF provide unique opportunities for this task. Utilizing the CyTOF approach, we analyzed the infiltrating immune cells from DA surgical tissues based on a panel of 33 markers and provided a single cell-resolution overview of the intricate DA immune microenvironment. Our study characterized the TIME in DAs, which is composed of a variety of immune cells, such as GAMs, CD8+ T cells, CD4+ T cells, Tregs, and NK cells. The enrichment of exhausted T cell subpopulations, recruitment of Tregs, and the strong pro-tumor phenotype of GAMs together contribute to the immunosuppressive microenvironment in DAs. DAs and OGs have been shown to share similar components and distributions of immune cells. However, the GAMs of DAs exhibit more inhibitory characteristics than those of OGs.

Historically, the central nervous system has been defined as “immunologically privileged” (34) and has been considered distinct relative to other organs due to the presence of the blood-brain barrier (BBB), which prevents the migration of immunocytes and cytokines into the brain (35). In LGG, the normal vascularization and the function of the BBB remain mostly intact and resemble that under normal conditions (36). In our study, the most abundant immune cells in DA were GAMs (70.02%) and T lymphocytes (20.86%). Compared with their counterparts in the paired PBMCs, the proportion of GAMs was significantly increased in DA lesions, while the proportions of T cells and B cells were significantly decreased, and the proportions of NK cells and granulocytes were similar. Our data suggest that although the BBB in DA lesions is fairly intact, certain immune cell populations can migrate across the BBB and infiltrate into the tumor, which might make them an adequate substrate for immunological antitumor therapies.

Inhibitory immune checkpoints are responsible for the dampening of antitumor immune functions (37). The development of immune checkpoint blockade therapies, including anti-PD-1 and anti-CTLA4 therapies, has provided new avenues for cancer treatment (38). Our results demonstrated that in the DA immune microenvironment, CD8+ T cell populations are highly enriched but express higher levels of PD-1 than those in the blood, and the expression level of antitumor-related factors is generally reduced. The increase in the quantity of exhausted CD8+ T cells in DA indicates that checkpoint blockade approaches that promote the antitumor effects of these immune cells may benefit immunotherapy of DA.

With the 2016 update of the WHO classification of tumors of the central nervous system (39), WHO grade II DA and OG tumors have been subcategorized according to distinct molecular markers. Patients with WHO grade II DAs and OGs were found to have statistically significant differences in progression-free survival (PFS), with OG patients having a statistically better PFS than DA patients (40). Little is known about how the microenvironment differs between DAs and OGs. Our study found that the immune cell composition of DA and OG was similar, and T cells in both diseases showed similar exhaustion characteristics. However, GAMs in DAs expressed higher levels of VEGF and TGFβ and exhibited more adverse immune-inhibitory characteristics than OGs.

Finally, while our study has presented useful resources and novel insights into the cellular composition and functions of the TIME in DAs, a limited number of cases have been collected in this pilot study. Future validation in a larger collection of patients would further support our conclusions and better characterize the prognostic values of immune components for DA.

## Data Availability Statement

The datasets generated for this study are available on request to the corresponding author.

## Ethics Statement

This study was approved by the Institutional Review Board and Ethics Committee of Beijing Tiantan Hospital, Capital Medical University. Written informed consent was obtained from each patient.

## Author Contributions

YC, SW, DC, JiangW, and JZ conceived and designed the study. WF and WW analyzed and interpreted the CyTOF data. HL, YJ, RH, JieW, JiancW, HX, and ZY participated in sample collection and data acquisition. All authors participated in the drafting of the manuscript, read and approved the final version of the manuscript, and gave their consent for publication.

### Conflict of Interest

The authors declare that the research was conducted in the absence of any commercial or financial relationships that could be construed as a potential conflict of interest.
