# Supplementary material for: High Dimensional Mass Cytometry Analysis Reveals Characteristics of the Immunosuppressive Microenvironment in Diffuse Astrocytomas
Source: Front Oncol. 2020 Feb 4;10:78. doi: 10.3389/fonc.2020.00078 (PMC7010913; doi:10.3389/fonc.2020.00078)
Supplement: Supplementary file 1 [file Table_1.DOCX]

**Table S1 Mass cytometry panel**

| **Marker** | **Metal isotope** | **Source** | **Catalog No.** | **Description** |
| --- | --- | --- | --- | --- |
| CD45 | 89Y | Fluidigm | 3089003B | Hematopoietic marker |
| CD45 | 156Gd | Fluidigm | 3156010B | Hematopoietic marker |
| CD3 | 147Sm | Fluidigm | 3147009B | T cells |
| CD4 | 145Nd | Fluidigm | 3145001B | CD4 T cells |
| CD8 | 146Nd | Fluidigm | 3146001B | CD8 T cells |
| CD19 | 150Nd | Biolegend | 302247 | B cells |
| CD66b | 162Dy | Fluidigm | 3162023B | Granulocytes |
| CD16 | 148Nd | Fluidigm | 3148004B | NK cells |
| CD56 | 155Gd | Fluidigm | 3155008B | NK cells |
| CD11b | 209Bi | Fluidigm | 3209003B | Macrophage/microglia marker |
| CD68 | 171Yb | Fluidigm | 3171011B | Glycoprotein |
| CD14 | 151Eu | Fluidigm | 3151009B | Monocyte marker |
| HLA-DR | 174Yb | Fluidigm | 3174001B | MHC class II molecule |
| CD206 | 168Er | Fluidigm | 3168008B | Mannose receptor |
| CD25 | 149Sm | Fluidigm | 3149010B | Tregs |
| CD127 | 176Yb | Fluidigm | 3176004B | Tregs |
| CD45RA | 143Nd | Fluidigm | 3143006B | T cell subset |
| CCR7 | 159Tb | Fluidigm | 3159003A | T cell subset |
| CD33 | 144Nd | Biolegend | 303302 | Myeloid marker |
| CXCR3 | 141Pr | Biolegend | 353733 | Chemokine receptor |
| CCR4 | 158Gd | Fluidigm | 3158032A | Chemokine receptor |
| PD-1 | 169Tm | Biolegend | 329941 | Checkpoint receptor |
| PD-L1 | 163Dy | Biolegend | 329719 | Checkpoint ligand |
| TIM-3 | 154Sm | Fluidigm | 3154010B | Checkpoint receptor |
| LAG-3 | 175Lu | Fluidigm | 3175033B | Checkpoint receptor |
| VEGF | 142Nd | Biolegend | 627501 | Cytokine |
| TGFβ | 153Eu | Biolegend | 349602 | Immunosuppressive cytokine |
| IDO | 160Gd | Biolegend | 695002 | Immune checkpoint protein |
| IL-10 | 166Er | Fluidigm | 3166008B | Immunosuppressive cytokine |
| IFNγ | 165Ho | Fluidigm | 3165002B | Cytokine |
| TNFα | 152Sm | Fluidigm | 3152002B | Cytokine |
| TNFβ | 172Yb | Biolegend | 503002 | Cytokine |
| Granzyme B | 173Yb | Fluidigm | 3173006B | cytotoxic serum protease protein |
| T-bet | 161Dy | Fluidigm | 3161014B | Transcription factor |
