# Supplementary material for: High Dimensional Mass Cytometry Analysis Reveals Characteristics of the Immunosuppressive Microenvironment in Diffuse Astrocytomas
Source: Front Oncol. 2020 Feb 4;10:78. doi: 10.3389/fonc.2020.00078 (PMC7010913; doi:10.3389/fonc.2020.00078)
Supplement: Supplementary file 2 [file Table_2.DOCX]

**Table S2 Cell type identification**

| **Immunocyte** | **Markers** |
| --- | --- |
| T cell | CD45+ CD3+ |
| CD4+ T cell | CD45+ CD3+ CD4+ |
| CD8+ T cell | CD45+ CD3+ CD8+ |
| B cell | CD45+ CD19+ |
| NK cell | CD45+ CD3- CD16+ CD56+ |
| Granulocyte | CD45+ CD66b+ |
| Monocyte | CD45+ CD14+CD16+ |
| Macrophages/Microglia | CD45+ CD11b+ CD3- CD19- CD66b- |
| Treg | CD45+ CD4+ CD25+ CD127- |
